# Lack of intracellular replication of *M. tuberculosis* and *M. bovis* BCG caused by delivering bacilli to lysosomes in murine brain microvascular endothelial cells

**DOI:** 10.18632/oncotarget.5932

**Published:** 2015-09-30

**Authors:** Xi Chen, Kaori Sakamoto, Frederick D. Quinn, Huanchun Chen, Zhenfang Fu

**Affiliations:** ^1^ State-key Laboratory of Agricultural Microbiology, College of Veterinary Medicine, Huazhong Agricultural University, Wuhan, China; ^2^ Department of Pathology, College of Veterinary Medicine, University of Georgia, Athens, GA, USA; ^3^ Department of Infectious Diseases, College of Veterinary Medicine, University of Georgia, Athens, GA, USA

**Keywords:** mycobacterium, endothelial cells, cytotoxicity, trafficking, cell-to-cell spread, Immunology and Microbiology Section, Immune response, Immunity

## Abstract

Invasion and traversal of the blood-brain barrier (BBB) by *Mycobacterium tuberculosis* cause meningeal tuberculosis (TB) in the central nervous system (CNS). Meningeal TB is a serious, often fatal disease that disproportionately affects young children. The mechanisms involved in CNS invasion by *M. tuberculosis* bacilli are poorly understood. In this study, we microscopically examined endosomal trafficking and measured survival of *M. tuberculosis* and *M. bovis* Bacille Calmette-Guérin (BCG) bacilli in murine brain microvascular endothelial cells (BMECs). The results show that both species internalize but do not replicate in BMECs in the absence of a cytotoxic response. Confocal microscopy indicates that bacilli-containing vacuoles are associated with the early endosomal marker, Rab5, late endosomal marker, Rab7, and lysosomal marker, LAMP2, suggesting that bacilli-containing endosomes mature into endolysosomes in BMECs. Our data also show that a subset of intracellular *M. tuberculosis*, but not BCG bacilli, escape into the cytoplasm to avoid rapid lysosomal killing. However, the intracellular mycobacteria examined cannot spread cell-to-cell in BMECs. Taken together, these data show that with the exception of the small terminal cytoplasmic population of bacilli, *M. tuberculosis* does not modulate intracellular trafficking in BMECs as occurs in macrophages and lung epithelial and endothelial cells.

## INTRODUCTION

Meningeal tuberculosis (MTB), caused by *Mycobacterium tuberculosis* (*Mtb*), is an often fatal form of TB and disproportionately affects children [1]. In order for meningitis to develop, *Mtb* bacilli must transit through the blood and traverse the blood-brain barrier (BBB), which is formed primarily by a tight monolayer of brain microvascular endothelial cells (BMECs) [2, 3]. Reports using mice intravenously inoculated with *Mtb* bacilli have reported that the bacteria can enter the central nervous system [4]. In an *in vitro* human BBB model, *Mtb* bacilli were shown to successfully invade and traverse the BMEC monolayer; host-cell actin cytoskeletal rearrangements were required for this process [5], but the cellular and molecular mechanisms involved are poorly understood.

It is hypothesized that the intracellular state provides a safe haven for a variety of pathogenic bacteria by limiting interactions between infectious disease agents and host cells. In the case of *Mtb*, bacilli avoid lysosomal fusion through the manipulation of host signal transduction pathways and the alteration of endocytic trafficking, resulting in privileged replicative niches [6]. For example, the late endosome marker, Rab7, but not lysosomal markers, LAMP2 and cathepsin L, rapidly accumulates in the mycobacterial endosomal membrane following the deposition of Rab5 in A549 human alveolar epithelial cells and endothelial cells [7, 8]. However, the early endosome marker, Rab5, but not the late endosome and lysosomal markers, accumulate in endosomal membranes containing the bacilli in macrophages [9]. In addition, the lack of acidification in the mycobacterial phagosome of macrophages is mainly due to the absence of lysosomal acid proteases, cathepsin, and/or vacuolar-ATPase (V-ATPase) on the phagosomal membranes [10, 11]. Non-pathogenic mycobacteria, such as *M. smegmatis*, cannot alter intracellular trafficking in macrophages and are degraded following phagosome-lysosome fusion [12].

It is also believed that release of the bacteria from these host cells is a means for dissemination to adjacent cells or distant tissues potentially *via* the blood and lymphatic systems [13]. *Listeria monocytogenes* and *Shigella flexneri* bacilli lyse the macrophage phagosomal membrane and escape into the host cytosol, where the bacteria replicate and spread to neighboring cells *via* actin-based motility [14]. Direct cell-to-cell spread allows these pathogens to continue to circumvent aspects of the humoral and cellular immune responses [6, 15]. Alternatively, *Mtb* bacilli are believed to exist exclusively within macrophage phagosomes [16], although this has recently been contested. However, like *L. monocytogenes* and *S. flexneri*, it has been shown that non-tuberculous mycobacteria infecting macrophages and *Mtb* bacilli infecting myeloid cells have the ability to translocate from the phagolysosome to the cytoplasm and *via* actin polymerization lead to direct cell-to-cell spread [17, 18]. This cytosolic translocation is reportedly an ESAT-6-dependent process, but thus far has not been demonstrated by *Mtb* bacilli in non-phagocytic cells [17, 19].

While BMECs are believed to play an important role during invasion and traversal of the BBB by *Mtb* bacilli, a thorough understanding of how the bacteria interact with the host cells to accomplish these tasks is lacking. The aim of this study was to examine the internalization, trafficking, and potential escape of *Mtb* bacilli in BMECs. Although this study showed that a majority of intracellular *Mtb* and *M. bovis* BCG bacilli are eliminated *via* traditional endosomal-lysosomal fusion processes, we did identify a subset of *Mtb* bacilli that escape to the cytoplasm and may serve as a basis for persistence in this cell type.

## RESULTS

### Internalization, but without intracellular replication of bacilli in BMECs

**Figure 1:**
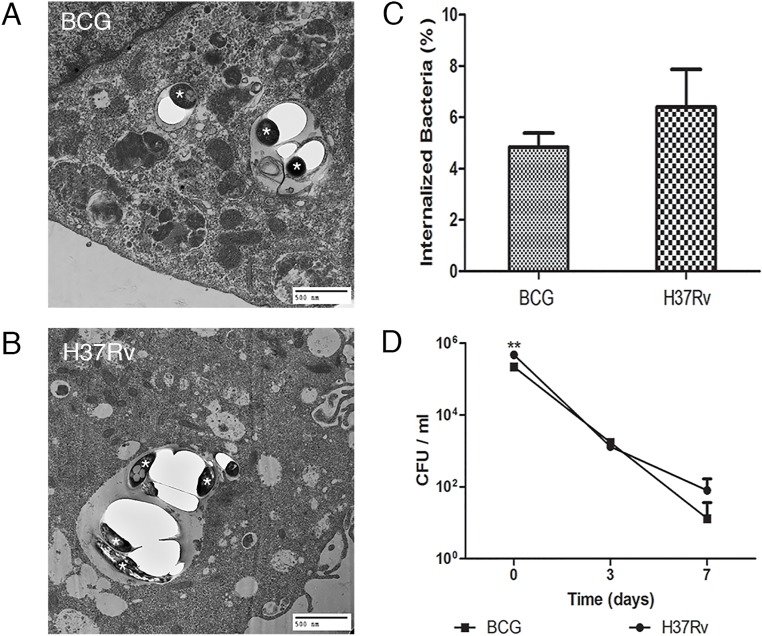
Both *Mtb* and BCG are similarly internalized by BMECs but do not replicate intracellularly Transmission electron microscopy (TEM) and bacterial viability measurements (CFU) were used to demonstrate internalization and survival of *Mtb* and BCG in BMECs. Mouse BMECs were infected with BCG **A.** or *Mtb* H37Rv **B.** for 6 h (day 0). The internalized bacilli (*) were within membrane-bound vacuoles. Initial internalized bacterial CFUs, as a percentage of the original inocula at day 0, showed no significant differences between the two species (*P* > 0.1) **C.** Over time, the number of intracellular bacteria from both species significantly decreased **D.** All experiments were performed in triplicate and repeated three times. Bars and points represent the mean, with error bars indicating the standard deviation.

*Mtb* and BCG bacilli were found in vacuoles of the endothelial cells by transmission electron microscopy (Figure 1A and 1B). CFU enumeration of *Mtb* and BCG bacilli showed 6.7% and 5.4% of the original inoculum were internalized and viable, respectively. Interestingly, there was no significant difference in CFU between the two species (*P* > 0.1) (Figure 1C). Analysis of up to 7 days postinfection showed the numbers of intracellular viable bacteria from both species significantly decreased (*P* < 0.001) (Figure 1D), suggesting that both species of *Mycobacterium* are not capable of intracellular replication in BMECs.

### Absence of cytotoxicity in infected BMECs

**Figure 2:**
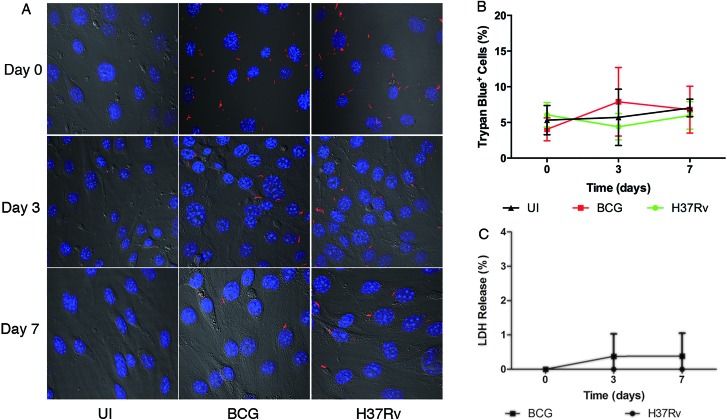
Confocal microscopy and cell viability assays demonstrate absence of cytotoxicity in infected BMECs Mouse BMECs were infected with fluorescent *Mtb* H37Rv or BCG bacilli for 6 h prior to washing and the addition of amikacin. Analyses were performed up to 7 days postinfection. A decreased number of bacteria associated with intact BMEC monolayers was observed from day 0 to day 7 **A.** Similar percentages of Trypan blue-positive, dead BMECs were counted in infected and uninfected monolayers **B.** For LDH detection, supernatants were collected at indicated time points and assayed using a cytotoxicity detection kit (Roche). A low percentage of LDH release was detected over time, and no significant differences were identified between the two species **C.** All assays were performed in triplicate and repeated three times. Confocal microscopy studies were performed at 600X. Data is reported from the average of three experiments. Error bars indicate standard deviations with *P* values determined by Student's *t*-test.

The failure of the examined mycobacteria to replicate inside the BMECs could be linked to a concomitant loss in host cell viability. To investigate this possibility, monolayers were infected with fluorescent *Mtb* or BCG bacilli and visualized by confocal microscopy. Neither *Mtb* nor BCG generated a disrupted phenotype in BMEC monolayers (Figure 2A). Lactate dehydrogenase (LDH) is a stable cytoplasmic enzyme and is rapidly released into the cell culture supernatant, when the host cell plasma membrane is damaged. To investigate whether mycobacterial infection induces a cytotoxic phenotype in BMECs, trypan blue viability staining and a LDH release assay were performed. No significant difference in percentage of trypan blue-positive cells was observed between cells infected with either *Mtb* or BCG, and uninfected cells (*P* > 0.1) (Figure 2B). In addition, no significant increase in LDH release was observed in cells infected with *Mtb* or BCG for up to 7 days (*P* > 0.1) (Figure 2C).

### Trafficking of bacilli into endolysosomes within BMECs

**Figure 3:**
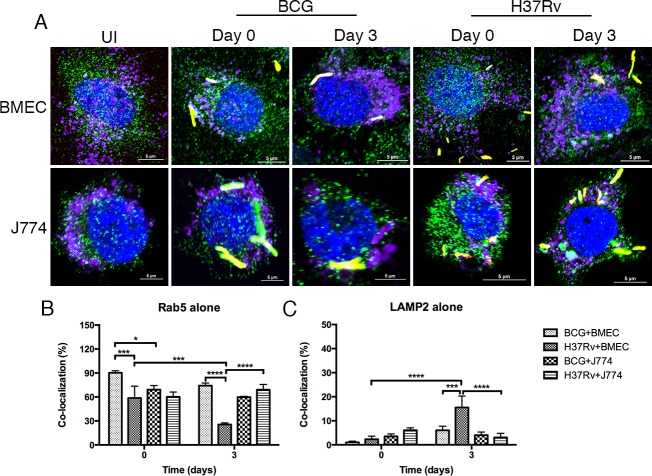
*Mtb-*containing vacuoles do not retain Rab5 or exclude LAMP2 in BMECs Microscopic examination of Rab5 or LAMP2 co-localization with *Mtb*- and *M. bovis* BCG-containing vacuoles in BMECs and J774A.1 cells. Mouse BMECs and J774A.1 cells were infected with fluorescent *Mtb* or *M. bovis* BCG bacilli (MOI = 10) and labeled with anti-Rab5 and anti-LAMP2 antibodies [Alexa Flour 488 (green) and Alexa Flour 647 (purple), respectively]. As shown in **A.**, fluorescent bacteria (red) are associated with Rab5 (green) and LAMP2 (purple)-positive compartments. Co-localization of bacteria with Rab5 and LAMP2 was quantified at the end of 6 h (day 0), and day 3 infections **B.** and **C.** Significant differences were observed between the different species and different time points (* *P* < 0.1; ** 0.001 < *P* < 0.01; *** 0.0001 < *P* < 0.001; **** *P* < 0.0001). A total of 200 bacteria from 20 fields in each specimen was analyzed. Infections were performed in triplicate and experiments repeated two times. Quantification is the average for all experiments. Error bars indicate standard deviations, with *p* values determined using one-way ANOVA followed by Tukey's test.

**Figure 4:**
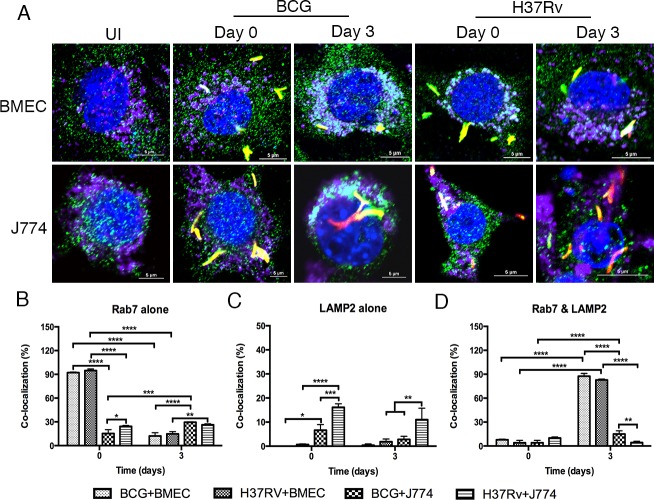
Both *Mtb-* and BCG-CVs retain Rab7 and LAMP2 together over time Microscopic examination of Rab7 and LAMP2 co-localization with *Mtb-* and BCG-CVs in BMECs and J774A.1 cells. Mouse BMECs and J774A.1 cells were infected with fluorescent *Mtb* or BCG (MOI = 10), and labeled with anti-Rab7 and anti-LAMP2 antibodies [Alexa Fluor 488 (green) and Alexa Fluor 647 (purple), respectively]. As shown in **A.**, fluorescent bacteria (red) are associated with Rab7 (green) and LAMP2 (purple). Co-localization of bacteria with Rab7 and LAMP2 was quantified at the end of 6 h (day 0), and day 3 infections **B.-D.** No significant differences were observed between the different species in BMECs; however, significant differences between time points are as indicated (* *P* < 0.1; ** 0.001 < *P* < 0.01; *** 0.0001 < *P* < 0.001; **** *P* < 0.0001). A total of 200 bacteria from 20 fields in each specimen was analyzed. Infections were performed in triplicate and experiments repeated two times. Quantification is the average for all experiments. Error bars indicate standard deviations, with *P* values determined using a one-way ANOVA followed by Tukey's test.

One of the strategies used by *Mtb* to survive and potentially replicate in macrophages and epithelial cells is to alter trafficking by inducing phagosomal maturation arrest (PMA) [7, 9]. Confocal studies have demonstrated that BMECs can be labeled for Rab GTPases [20]. To determine whether bacilli can induce PMA in BMECs, monolayers were infected with fluorescent *Mtb* or BCG. The association of early and late endosomal markers (Rab5 and Rab7) with mycobacteria-containing vacuoles (MCVs) was determined by confocal microscopy. As shown in Figure 3A and Figure 4A, both *Mtb*- and BCG-CVs are associated with Rab5 and Rab7. *Mtb*-CVs were 58.7% labeled with Rab5 alone at day 0 and then decreased to 25.5% by day 3 postinfection, whereas BCG-CVs maintained high levels of staining with Rab5 (Figure 3B). However, colocalization of *Mtb*- and BCG-CVs with Rab7 alone was significantly decreased from 94.8% and 92.2% at day 0 to 14.8% and 12.2% at day 3 postinfection respectively, with no significant difference between the two species of *Mycobacterium* (*P* > 0.1) (Figure 4B). As the positive control, *Mtb*- and BCG-CVs in J774A.1 cells maintained a high level (60.2% and 69.2% at day 0; 69% and 59.8% at day 3 postinfection respectively) of association with Rab5 alone (Figure 3B), but a low level (24.3% and 15.3% at day 0; 26.3% and 29.5% at day 3 postinfection, respectively) of association with Rab7 alone over time (Figure 4B). Both uninfected BMEC and J774 cells can be labeled with Rab5, Rab7 or LAMP2 with no difference at different time points.

To determine whether MCVs recruit lysosomal markers, cells were also immunostained for LAMP2, and the associations with other endosomal and/or lysosomal markers were quantified. In contrast to the J774A.1 control, *Mtb*- and BCG-CVs that labeled with both Rab7 and LAMP2 increased from 4% and 7.8% at day 0 to 82.8% and 87.5% at day 3 postinfection, respectively, with no significant differences between the two species (*P* > 0.1) (Figure 4D). In contrast to BCG-CVs and the uninfected J774A.1 control, *Mtb*-CVs that labeled with LAMP2 alone were significantly increased from 2.3% at day 0 to 15.5% at day 3 postinfection when also immunostaining for Rab5 (Figure 3C), but not Rab7 (Figure 4C). These data showed decreased numbers of bacilli associated with endosomes and increased numbers associated with endo/lysosomes over time, suggesting that both BCG- and *Mtb*-CVs fuse with lysosomes.

**Figure 5:**
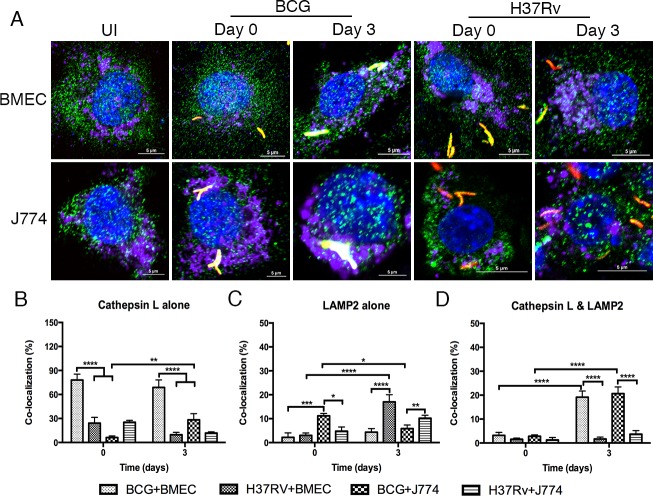
*Mtb-*CVs do not accumulate appreciable amounts of cathepsin L, relative to BCG Microscopic examination of cathepsin L and LAMP2 co-localization with *Mtb*- and BCG-CVs in BMECs and J774A.1 cells. Mouse BMECs and J774A.1 cells were infected with fluorescent *Mtb* or BCG bacilli (MOI = 10), and labeled with anti-cathepsin L and anti-LAMP2 antibodies [Alexa Flour 488 (green) and Alexa Flour 647 (purple), respectively]. As shown in **A.**, fluorescent bacteria (red) are associated with cathepsin L (green) and LAMP2 (purple). Co-localization of bacteria with cathepsin L and LAMP2 was quantified at the end of 6 h (day 0), and day 3 infections **B.-D.** A significant difference was observed between the different species and different time points (* *P* < 0.1; ** 0.001 < *P* < 0.01; *** 0.0001 < *P* < 0.001; **** *P* < 0.0001). A total of 200 bacteria from 20 fields in each specimen was analyzed. Infections were performed in triplicate and experiments repeated two times. Quantification is the average for all experiments. Error bars indicate standard deviations, with *p* values determined by one-way ANOVA followed by Tukey's test.

As shown in Figure 5A, both BCG- and *Mtb*-CVs are associated with the lysosomal enzyme, cathepsin L. BCG-CVs maintained high levels of association with cathpsin L alone (78% at day 0 and 68.8% at day 3 postinfection). Alternatively, *Mtb*-CVs (24.2% at day 0 and 9.7% at day 3 postinfection) or *Mtb*- and BCG-CVs in J774.1 cells (25.3% and 6.2% at day 0; 11.8% and 28.3% at day 3 postinfection respectively) maintained low levels of association with cathepsin L alone over time, with a significant difference between the two species (*P* < 0.001) (Figure 5B). In contrast to *Mtb*-CVs in BMECs or in J774.1 cells, BCG-CVs were 3.2% and 2.8% labeled with both cathepsin L and LAMP2 at day 0, which significantly increased to 19.2% and 20.7% at day 3 postinfection in BMECs and J774 cells, respectively (Figure 5D). Also, a significantly increased co-localization of *Mtb*-CVs in BMECs with LAMP2 alone when also immunostaining for cathepsin L, was observed at day 3 postinfection (Figure 5C), which supports our earlier findings. It is suggested that both BCG- and *Mtb*-CVs fuse with lysosomes, but *Mtb*-CVs have a limited association with cathepsin L. Taken together, the data further support that both *Mtb* and BCG traffic to lysosomal compartments.

### Translocation of virulent *Mtb* bacilli into the cytoplasm

**Figure 6:**
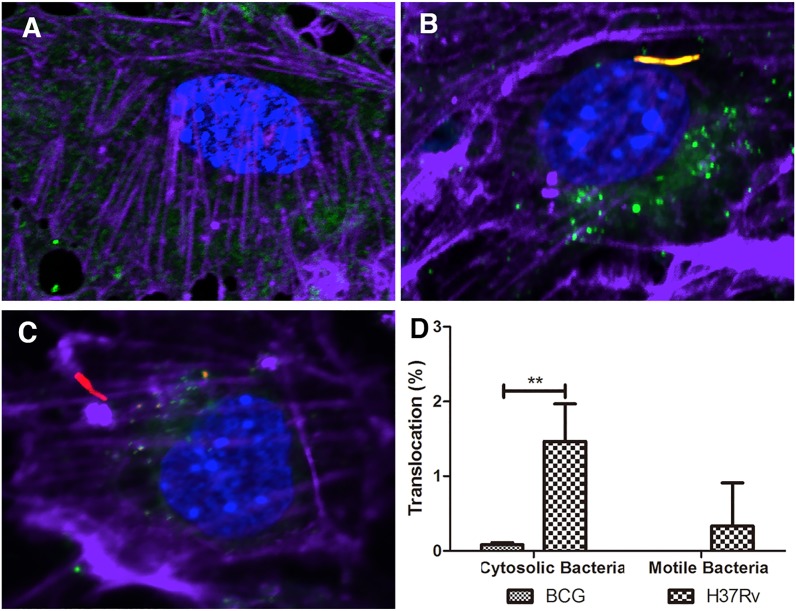
*Mtb* bacilli translocate into the cytosol of BMECs Mouse BMECs were infected with fluorescent *Mtb* or BCG (MOI = 10). The samples were stained with SP-DiOC_18_ (green), and Alexa Fluor 647 Phalloidin (purple). Uninfected BMECs were used as control **A.** The cytosolic bacteria and motile bacteria, as determined by the absence of co-localization with SP-DiOC_18_ and presence of an actin tail, were quantified. Less than 2% of intracellular *Mtb* bacilli could translocate into the cytosol, and a significant difference was observed between BCG and *Mtb* **B.-D.** (** 0.001 < *P* < 0.01). Very few cytosolic *Mtb* bacilli recruited actin tails **D.** A total of 200 bacteria from 20 fields were counted for each slide. Infections were performed in triplicate and experiments repeated two times. Quantification is the average for all experiments. Error bars indicate standard deviations with *P* values determined by Student's *t*-test.

Stamm *et al*. (2003) reported that *Mtb* bacilli are able to escape from phagosomes to the cytoplasm, where they polymerize actin “tails,” allowing mobility, and potentially, cell-to-cell spread. To investigate whether these mycobacteria translocate to the cytoplasm in BMECs, infected cells were stained with Alexa Fluor 647 Phalloidin and SP-DiOC_18_. As shown in Figure 6C, *Mtb* bacilli could translocate into the cytoplasm of BMECs, and a low percentage of cytosolic *Mtb* bacilli formed actin “tails,” but this did not occur in cells infected with BCG (Figure 6B and 6D). These results suggest that a small number of intracellular *Mtb* bacilli are able to escape the endolysosome into the cytoplasm; however, most of the cytosolic bacteria are not motile *via* actin polymerization. The ultimate fate of these cytoplasmic survivors is under investigation.

### Mycobacterial infection fails to develop microcolonies within BMECs

**Figure 7:**
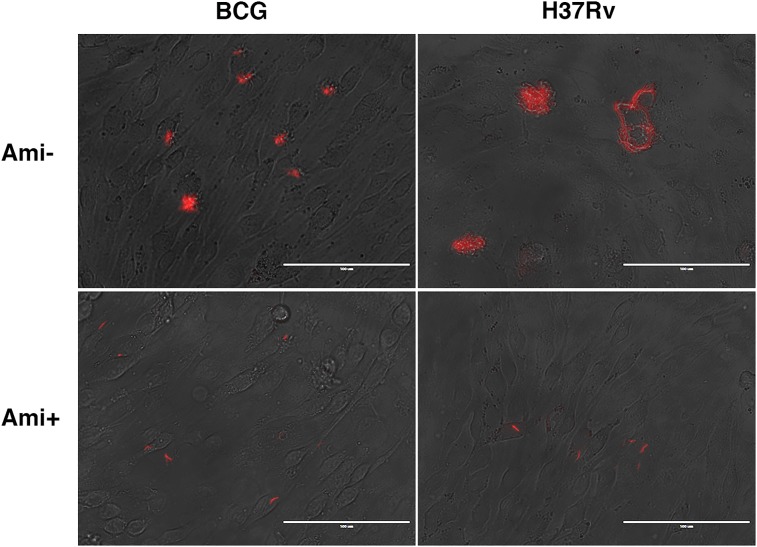
*Mtb* and BCG bacilli cannot spread between BMECs in a monolayer Cells were infected with fluorescent *Mtb* or BCG bacilli (MOI = 0.1) for 24 h, then washed three times with PBS to remove unbound bacteria. The cells were overlaid with 1% agarose/DMEM with 1% FBS, with or without amikacin (50 μg/ml) and cultured for up to 7 days. Extracellular bacterial replication was observed in amikacin-free infections with *Mtb* and BCG. However, plaque formation, indicating bacterial spread and cytotoxicity, was only found in *Mtb*-infected cells in the absence of amikacin. Infections were performed in triplicate and experiments repeated two times.

To investigate whether the cytosolic bacteria traffic out of cells and infect neighboring cells, a BMEC-mycobacterial microcolony assay was performed. Seven days after infection, fluorescent bacterial foci were visualized in fluorescent *Mtb*- or BCG-infected monolayers, and a disrupted monolayer infected with *Mtb* was observed in the absence of amikacin (Figure 7). To ensure that the small foci were formed by cell-to-cell spread, amikacin (50 μg/ml) was added to the tissue culture medium. Plaque formation was observed in monolayers without amikacin, but not observed up to day 7 in the presence of amikacin (Figure 7). These data suggest that BMEC monolayer disruption is associated with extracellular bacterial replication, and intracellular mycobacteria could not spread cell-to-cell. Also, this further confirms that *Mtb* and BCG bacilli cannot replicate intracellularly.

## DISCUSSION

In humans, meningeal TB is the result of traversal of *Mtb* bacilli from the lungs to the blood and ultimately to the brain and central nervous system. Although mice are generally seen as a poor model for TB pathogenesis studies, meningeal TB can develop in mice infected with *Mtb* bacilli [4, 21], as seen in humans. Respiratory infection or intravenous inoculation of *Mtb* bacilli in the murine model simulates disseminated hematogenous TB and produced meningitis, but the neuroparenchyma lacks such an immune response, which is consistent with human data [4, 21]. To penetrate the brain, *Mtb* bacilli have to cross the blood-brain barrier. It was previously reported that *Mtb* bacilli invade and traverse the BMEC monolayer [5], but the mechanism used by the bacilli to traffic within BMECs and traverse through the monolayer remains unknown. Basing our hypothesis on the interaction of *Mtb* bacilli and human lung endothelial cells [22], we examined whether these bacilli traverse the BBB by internalization, intracellular replication, and cytotoxic destruction of the host cell, thus breaking the barrier. However, our data showed that bacilli internalize but lack the ability to replicate intracellularly, effect cytotoxicity, or spread from cell-to-cell.

In terms of their antimicrobial capacity, we determined that the BMECs can restrict the intracellular growth of both tested species of *Mycobacterium* by delivering them to lysosomal compartments for degradation. Both endosomal and lysosomal markers accumulate on MCVs, indicating that endosomes containing the examined *Mycobacteria* mature into endolysosomes. Interestingly, MCVs containing *Mtb*, but not BCG, were weakly positive for the lysosomal enzyme, cathepsin L, suggesting *Mtb* bacilli could reside in endolysosomes, potentially surviving longer intracellularly for reasons that are currently unclear. In contrast to BMECs, *Mtb* bacilli are capable of replicating in human lung microvascular endothelial cells (HULECs), human type II alveolar epithelial cells (A549s), and non-activated macrophages [22-24]. This capacity by *Mtb* to modulate intracellular trafficking in these cell types likely supports intracellular survival and replication.

The intracellular replication of *Mtb* bacilli is cytotoxic for A549 cells by inducing necrosis and membrane permeability enhancements that may contribute to the dissemination of the bacilli across the epithelial barrier and into the circulatory system [25, 26]. However, Castro-Garza *et al*. (2012) reported that this cytotoxicity is not totally dependent on intracellular growth but also correlates with strain virulence [27]. In our study, we found that intracellular persistence of *Mtb* bacilli did not generate a cytotoxic response, and the BMECs did not detach from the monolayer, thus an as yet unknown mechanism for dissemination from BMECs is at work.

The traversal of BBB by *L. monocytogenes* is mediated by cell-to-cell spread. This ability may shield this organism from immune responses, particularly those involving antibody or complement, and could provide a safe haven for long-term persistence [15]. Our data show that a subset of *Mtb* bacilli could escape into the cytosol and recruit actin, but could not traffic out of the cell in the examined time frame to infect adjacent cells. Other mycobacteria have been shown to be able to escape into the cytoplasm of infected cells, where they recruit host cell cytoskeletal factors to induce actin polymerization leading to direct cell-to-cell spread [17, 18]. The translocation of the bacilli into the cytoplasm is dependent on the type VII ESX-1 secretion system, which secretes early-secreted antigen 6 kDa (ESAT-6) and culture filtrate protein 10 KDa (CFP-10). The C-terminus of the ESAT-6 facilitates the escape of mycobacteria from the vacuoles by producing pores in MCV membranes [19, 28].

Although the mechanism of traversal of the BBB by *Mtb* is still unclear, a tissue-specific gene, *pknD*, was found to be sufficient to trigger the internalization of *Mtb* bacilli into BMECs, and bacterial dissemination to the brain was attenuated in recombinant *Mtb* PknD-vaccinated animals [29, 30]. Infection by *Mtb* bacilli did not cause any noticeable lysis or detachment of the BMEC monolayer at the end of the invasion and traversal assay in an *in vitro* model [5]. However, the BBB disruption detected in TB meningitis was shown to be related to vascular endothelial growth factor (VEGF) and tumor necrosis factor alpha (TNF-α) [31, 32]. Future experimentation will examine the effects of these factors on the infection and dissemination process in our BMEC system.

## MATERIALS AND METHODS

### Bacterial culture

*Mtb* H37Rv and *M. bovis* Bacille Calmette-Guérin (BCG, Pasteur) bacilli were grown in Middlebrook 7H9 liquid broth (Becton Dickinson) supplemented with 0.5% glycerol, 0.05% Tween-80, and 10% oleic acid albumin dextrose catalase (OADC, Becton Dickinson). For confocal microscopy, bacilli were transformed with a plasmid expressing DsRed2 and maintained by inclusion of hygromycin B (50 μg/ml). For infection, the bacterial culture optical densities at 600 nm was adjusted to achieve the required MOI and centrifuged at 1,000 g for 10 min to pellet the bacteria. The pellet was resuspended in infection medium and passed through an insulin syringe to disperse the bacteria. In addition, 50 μl from each serially-diluted inocula were plated to determine the number of viable bacteria (colony forming units - CFU).

### Cell culture

Mouse BMECs (bEnd.3, ATCC^®^ CRL-2299) and macrophages (J774A.1, ATCC^®^ TIB-67) were maintained in Dulbecco's Modified Eagle's Medium (DMEM) supplemented with 10% fetal bovine serum (FBS) (ATCC). For confocal microscopy, 5.0 × 10^4^ cells were seeded onto cover slips coated with collagen I (6 μg/cm^2^) in a 24-well plate and incubated for 24 h at 37°C in 5% CO_2_.

### Initial internalization assay

Bacterial suspension (MOI = 10) was applied to each well for 6 h at 37°C in 5% CO_2_. The cells were washed with pre-warmed PBS to remove unbound bacteria, and incubated with amikacin (200 μg/ml) for 2 h to kill remaining extracellular mycobacteria. The cells were washed with pre-warmed PBS and then lysed using sterile 0.1% Tween 80 in water for 10 min at 37°C. CFUs were determined to quantify intracellular bacteria. Internalization was expressed as a percentage of intracellular bacteria compared to the inoculum.

### Intracellular bacterial viability assay

Cells were incubated with bacilli (MOI = 10) for 6 h at 37°C in 5% CO_2_. The cells were washed with pre-warmed PBS, and supplied with fresh DMEM with 10% FBS containing amikacin (50 μg/ml) (referred to as day 0). The medium was changed every two days to avoid serum starvation and death of mycobacteria due to autophagy induction. The infected cells were lysed at indicated time points using sterile 0.1% Tween 80 in water, and viable bacilli were enumerated by serial dilution of lysate and plating as described above. All infections were performed in triplicate and the experiment repeated at least three times.

### Transmission electron microscopy

Cells (3×10^5^ per well) were seeded into a 6-well plate, and infected for 6 h (MOI = 10). The infected cells were fixed overnight with 2% paraformaldehyde, 2% glutaraldehyde, and 0.2% picric acid in 0.1 M cacodylate-HCl buffer (pH 7.25). After rinsing for 15 min at least 3 times in 0.1 M cacodylate-HCl buffer, the cells were post-fixed in 1% OsO_4_/0.1 cacodylate-HCl buffer, and stained with 0.5% uranyl acetate (aqueous) for 1 h after rinsing with deionized water. An ethanol series was used to dehydrate the specimens. Thorough infiltration was completed with three ratios of prolylene oxide: resin (Epon-araldite). Samples were embedded and polymerized for 18-24 h at 60-70°C. Ultrathin sections were mounted onto copper grids and stained with 4% uranyl acetate and lead citrate. Imaging was performed using a JEM-1210 Transmission Electron Microscope (JEOL, Tokyo, Japan) operating at 120 kV. Digital images were captured using a XR41C Bottom-Mount CCD Camera (AMT, Danvers, MA).

### Confocal microscopy

For visualization, the infected cells were fixed with 4% paraformaldehyde overnight at 4°C. Specimens were washed with PBS and mounted onto microscope slides using Prolong antifade reagent with DAPI (Invitrogen).

For trafficking assays, the infected cells were fixed at indicated time points with 4% paraformaldehyde. After blocking with 5% normal goat serum in 0.3% Triton X-100 PBS for 1 h at room temperature, the cells were incubated with a 1:300 dilution of anti-Rab5 (Abcam), anti-Rab7 (Santa Cruz Biotechnology), anti-LAMP2 (Abcam), or anti-cathepsin L (Abcam) antibody for 2 h at room temperature. Alexa Fluor^®^ 488 goat anti-rabbit IgG (Invitrogen) was used at a 1:600 dilution to detect LAMP2, and Alexa Fluor^®^ 647 goat anti-rat IgG (Santa Cruz Biotechnology) was used at a 1:300 dilution to detect Rab5, Rab7, and cathepsin L. The coverslips were incubated with a 1:5,000 dilution of DAPI (Invitrogen) in PBS for 5 min and mounted onto microscope slides using Prolong antifade reagent (Invitrogen).

For translocation assays, the cells were incubated with 2 μM SP-DioC_18_ (Invitrogen) to label intracellular membranes for 20 min at 37°C before fixation at day 3 postinfection. The infected cells were fixed with 4% paraformaldehyde, and permeabilized with 0.1% Triton X-100 for 1 h at room temperature. Alexa Fluor^®^ 647 phalloidin (Invitrogen) was added to specimens for 1 h at room temperature to stain for F-actin. DAPI in PBS (1:5,000) was applied to specimens for 5 min followed by washing three times with PBS. The specimens were mounted onto microscope slides using Prolong antifade reagent (Invitrogen).

All infections were performed in triplicate, and experiments were repeated two times. Two hundred bacteria in total were analyzed for each assay. Images were obtained with a Nikon A1R confocal laser microscope system equipped with NIS-Elements Imaging Software (Version 4.13; Nikon Instruments Inc. Melville. NY).

### Lactate dehydrogenase release assay and cell counts

Supernatants were collected at indicated time points, and passed through polyvinylidene fluoride (PVDF) membranes (0.22 μm pore size). For a maximum release control, Triton X-100 (final concentration is 0.02%) was used to lyse cells. Immediately following collection, supernatants were assayed for LDH activity using the Cytotoxicity Detection Kit per manufacturer instructions (Roche, Indianapolis, IN, USA). Percent LDH release was calculated using the following formula: [(release from infected cells - Background) / (Max release - Background)] × 100.

For cell counts, monolayers were washed with PBS, and then treated with 0.25% (w/v) Trypin-0.03% (w/v) EDTA solution (Gibco) for 5 min at 37°C. Live and dead cells were counted after staining with 0.4% trypan blue solution. Percent dead cells were calculated using the following formula: trypan blue positive cells / total cells × 100. All infections were performed in triplicate, and experiments were repeated three times.

### BMEC microcolony assay

Cells (3×10^5^ per well) were seeded into six-well plates, and incubated in 5% CO_2_ at 37°C until ≥ 90% confluence (~2.0 × 10^6^ cells). Bacteria suspension (MOI = 0.1) was applied to the well for 24 h at 37°C in 5% CO_2_. The monolayers were washed with PBS and overlaid with 2 ml of DMEM with 1% FBS containing 1% agarose (Invitrogen) (cooled to 42°C). In some wells, culture medium containing amikacin (50 μg/ml) was added. To maintain a sufficient antibiotic concentration in the medium and sufficient nutrition, 1 ml of fresh DMEM with 1% FBS with or without amikacin was supplied every 2 days. The infected cultures were observed for plaque formation at day 7 under a light and fluorescence microscope.

### Statistical analysis

Data were analyzed and plotted using Graphpad Prism 5.0 (La Jolla, CA). Data are expressed as mean ± standard deviation. Evaluation of the significance of differences between groups was performed by using the Student *t*-test or one-way ANOVA followed by Tukey's test as noted in figure legends.
